# Neuroprotective Effects of Ethanol Extract *Polyscias guilfoylei* (EEPG) Against Glutamate Induced Neurotoxicity in HT22 Cells

**DOI:** 10.3390/ijms252212153

**Published:** 2024-11-12

**Authors:** Qui Ngoc Sang Nguyen, Ki-Yeon Yoo, Thi Thu Trang Pham, Baskar Selvaraj, Huong Thuy Vu, Tam Thi Le, Heesu Lee, Quang Luc Tran, Phuong Thien Thuong, Ae Nim Pae, Sang Hoon Jung, Jae Wook Lee

**Affiliations:** 1 Natural Product Research Center, Institute of Natural Products, Korea Institute of Science and Technology, Gangneung 25451, Republic of Korea; kevindh2515@kist.re.kr (Q.N.S.N.); 523502@kist.re.kr (T.T.T.P.); sbaskar@kist.re.kr (B.S.); ttle@kist.re.kr (T.T.L.); 2 Department of Anatomy, College of Dentistry and Research Institute for Dental Engineering, Gangneung Wonju National University, 7 Jukheon-gil, Gangneung 25457, Republic of Korea; kyyoo@gwnu.ac.kr (K.-Y.Y.); nightsu@gwnu.ac.kr (H.L.); 3 Institute of Natural Product Chemistry, Vietnamese Academy Science and Technology, 1H Building, 18 Hoang Quoc Viet Street, Cau Giay, Hanoi 100000, Vietnam; 4 Natural Product Applied Science, KIST School, University of Science and Technology (UST), Gangneung 25451, Republic of Korea; 5 Traphaco Join-Stock Company, 75 P. Yên Ninh, Quán Thánh, Ba Đình, Hanoi 1000000, Vietnam; vhthuy111@gmail.com (H.T.V.); luctq@traphaco.com.vn (Q.L.T.); 6 Faculty of Herbal Medicine, Traditional Pharmacy, Hanoi University of Pharmacy, 13-15 Le Thanh Tong, Ba Dinh, Hanoi 100000, Vietnam; 7 Division of Biotechnology, Vietnam Korea Institute of Science and Technology, Hoa lac High-tech Park, km29 Thang Long Boulevard, Hanoi 100000, Vietnam; ptthuong.vkist@gmail.com; 8 Division of Bio-Medical Science & Technology, KIST School, Korea University of Science and Technology (UST), Seoul 02792, Republic of Korea; anpae@kist.re.kr; 9 Center for Brain Disorders, Brain Research Institute, Korea Institute of Science and Technology, Seoul 02792, Republic of Korea

**Keywords:** *Polyscias guilfoylei*, HT22 cells, neuroprotection, ischemic brain injury

## Abstract

Oxidative stress induced by glutamate is a significant contributor to neuronal cell damage and can lead to neurodegenerative diseases such as Alzheimer’s, Huntington’s, and ischemic brain injury. At the cellular level, oxidative stress increases Ca^2+^ ion influx and reactive oxygen species (ROS), which activate the MAPK signaling pathway. Additionally, the generation of ROS causes mitochondrial dysfunction, triggering apoptosis by promoting the translocation of AIF to the nucleus from the mitochondria. The neuroprotective potential of *Polyscias guilfoylei* has not yet been reported. Therefore, in this study, the ethanol extract of *Polyscias guilfoylei* (EEPG) was examined for its protective effect against oxidative cell damage caused by glutamate in neuronal cells. EEPG treatment increased the viability of HT22 cells exposed to high concentrations of glutamate. Cellular Ca^2+^ ion influx and ROS generation decreased with EEPG treatment in glutamate-treated HT22 cells. EEPG treatment inhibited MAPK activation and AIF nuclear translocation. In an in vivo study, EEPG attenuated brain cell death in an ischemic brain injury rat model. This study demonstrates the potential therapeutic effects of *Polyscias guilfoylei* in the treatment of ischemic brain injury.

## 1. Introduction

The incidence of neurodegenerative diseases is gradually increasing due to the increasing number of the elderly population. As a result, growing societal concern about neurodegenerative diseases has increased the demand for various therapeutic approaches to prevent the onset of these conditions. Neurodegenerative diseases are caused by the progressive loss of a relatively vulnerable population of neurons [1]. Although the loss or malfunction of neurons is regarded as a major causative factor in neurodegenerative diseases, the molecular mechanisms underlying neuronal loss or malfunction are not yet fully understood. Glutamate is the primary stimulatory neurotransmitter that plays a key role in various functions, including memory, cognition, and mood regulation [2]. High concentrations of glutamate in the synapse cause neurotoxicity by elevating intracellular levels of ROS [3,4]. ROS is mainly implicated in neuronal cell death, which results in Alzheimer’s [5,6,7,8], Parkinson’s [9,10,11], and Huntington’s diseases [12,13].

At the cellular level, excess glutamate blocks the cysteine/glutamate antiporter, which normally transports cysteine into cells. Consequently, the depletion of cellular cysteine leads to a reduction in the production of the cellular antioxidant glutathione, leading to the accumulation of intracellular ROS [14]. The reduction in glutathione levels can activate various signaling pathways that lead to apoptosis by increasing cellular Ca^2+^ ion influx and peroxidation of lipids. Increased cellular Ca^2+^ influx activates the enzyme calpain, which cleaves the pro-apoptotic protein Bid to tBid. This can activate the pro-apoptotic protein Bax [15]. Oxidative stress causes mitochondrial dysfunction, which can induce the AIF (apoptosis-inducing factor) which translocates to the nucleus from mitochondria. AIF nuclear translocation can induce a caspase-independent apoptotic pathway [16].

Meanwhile, oxidative stress can trigger a cascade by phosphorylating mitogen-activated protein kinases (MAPKs), such as extracellular signal-regulated kinase (ERK), c-Jun N-terminal kinase (JNK), and p38 MAPK [17,18,19]. The oxidative stress-mediated activation of the MAPK pathway is linked to the activation of cell death [20].

Medicinal plant extracts are considered a safe and promising alternative approach for treating diseases because specific medicinal plants have been used for disease treatment for a long time. Therefore, the safety of medicinal plants has been well established. *Polyscias guilfoylei* belongs to the Araliaceae family and its slow-growing ornamental plant is distributed in Southeast Asia and the Pacific areas, including Vietnam. *Polyscias* species have been used for pharmaceutical purposes in Vietnam for a long time, leading to several reports on their chemical constituents. Chemical components isolated from Polyscias guilfoylei have demonstrated various bioactivities, including cytotoxic effects, antibacterial activity, and inhibition of histamine release [21]. However, the neuroprotective capabilities of *Polyscias guilfoylei* have not been explored so far. Therefore, we have investigated the neuroprotective potential, mode of action, and chemical constituents of the ethanol extract of *Polyscias guilfoylei* (EEPG). Furthermore, we have studied the in vivo efficacy of EEPG in an ischemic brain injury animal model.

## 2. Results

### 2.1. Protective Effects of Ethanol Extracts of Polyscias guilfoyei (EEPG) on Glutamate Induced HT22 Cells

**Figure 1:**
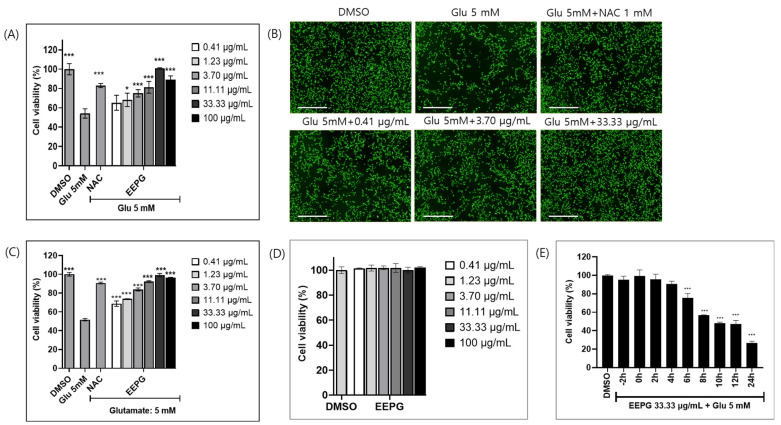
Neuroprotective effect of EEPG and its cell viability result. (**A**) Image analysis using Calcein AM confirms the neuroprotective effects of EEPG. The treatment between 11.33 μg/mL and 100 μg/mL of EEPG showed more than 80% neuroprotective effect to glutamate-induced cytotoxicity. (**B**) These cells were stained with Calcein AM. Calcein AM selectively stained live cells. In image analysis, 33.33 μg/mL of EEPG shows potent neuroprotective effects in HT22 cells (Scale bars = 1 mm). (**C**) Ez-cytox assay reveals that the treatment of EEPG at a concentration range between 3.70 μg/mL and 100 μg/mL showed potent neuroprotective effects in glutamate-induced cytotoxicity. (**D**) The cytotoxicity of EEPG. EEPG shows no cytotoxicity at 100 μg/mL. (**E**) The cell survival of HT22 cells for 24 h after incubation of 5 mM of glu. EEPG was added at the indicated times before and after the addition of 5 mM of glu. *** *p* < 0.001 and * *p* < 0.02 versus 5 mM glu alone group.

To elucidate the neuroprotective effects of the EEPG, we first determined the glutamate concentration that activates oxidative stress in HT22 cells. In this experiment, we found that 5 mM of glutamate and 3000 cells per well are the optimal conditions for the neuroprotective assay (Figure S1). Using these conditions, we evaluated the neuronal cell protective effect of the *Polyscias guilfoyei* extract in HT22 cells using glutamate. Glutamate neurotoxicity led to the reduction in cell viability by 50% in 12 h treatment. In total, 1 mM of NAC (N-acetyl cysteine) was used as a positive control. Co-treatment with EEPG prevented glutamate-induced cell death, resulting in 80% cell viability when EEPG was used at concentrations ranging from 3.70 μg/mL to 33.33 μg/mL (Figure 1A,B). This study is the first to demonstrate the neuroprotective properties of EEPG. Additionally, the neuroprotective effect of EEPG (ranging from 11.11 μg/mL to 100 μg/mL) was comparable to, or slightly stronger than, that of 1 mM NAC, indicating promising neuroprotective potential.

The neuroprotective effect of EEPG was also assessed using the Ez-Cytox cell viability assay. Co-treatment with EEPG and glutamate was performed, and after 12 h, the Ez-Cytox reagent was added, followed by measurement of absorbance at 450 nm. The results of this assay also indicated that EEPG treatment exhibits neuroprotective effects at concentrations between 3.70 μg/mL and 33.33 μg/mL, consistent with the results obtained using the calcein AM assay. These data clearly demonstrate that EEPG pretreatment provides neuroprotection against glutamate (Figure 1C). The toxicity of EEPG was evaluated in HT22 cells, and treatment with 100 μg/mL of EEPG did not exhibit significant cytotoxicity in HT22 cells (Figure 1D).

To measure the neuroprotective effects of EEPG post-treatment, glutamate was applied first, followed by EEPG treatment at the indicated time points up to 24 h to assess its protective effects after glutamate exposure. The results indicated that EEPG administered within 6 h after glutamate exposure prevented glutamate-induced neurotoxicity (Figure 1E). Therefore, we further explored the molecular mechanisms responsive to the neuroprotective effects of EEPG.

### 2.2. EEPG Attenuated Cellular Ca^2+^ Concentration, ROS Generation, and MAPK Activation in Glutamate-Induced HT22 Cells

**Figure 2:**
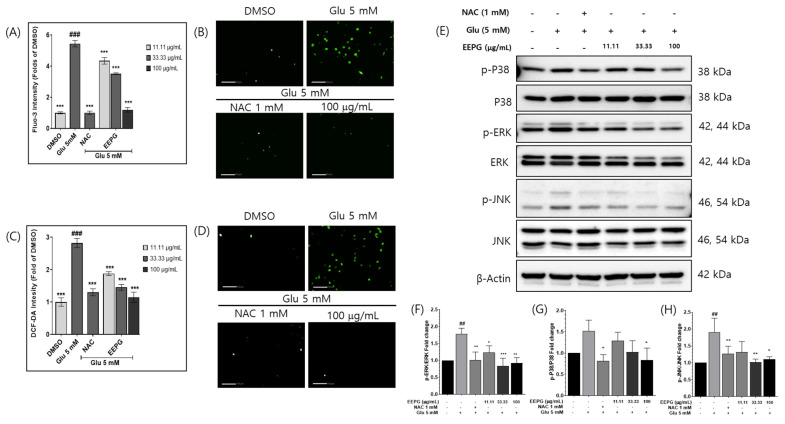
(**A**) The bar graph shows the effects of EEPG on Ca^2+^ ion concentration. An amount of 5 mM of glutamate treatment increases cellular Ca^2+^ ion concentration. Cotreatment of EEPG and glutamate decreases cellular Ca^2+^ ion concentration. (**B**) The image of cellular Ca^2+^ ion. The image was generated using a Flu-3 fluorescent Ca^2+^ ion sensor. 5 mM glutamate increased fluorescent intensity of Ca^2+^ ion sensor. Co-treatment with 100 μg/mL EEPG decreased fluorescent intensity of Ca^2+^ ion sensor. Scale bars = 100 μm. (**C**) The bar graph shows the inhibitory effect of EEPG on ROS. (**D**) The image of ROS generated using a DCF-DA fluorescent ROS sensor. 5 mM glutamate increased fluorescent intensity of ROS sensor. Co-treatment with 100 μg/mL EEPG decreased fluorescent intensity of ROS sensor. Scale bars = 100 μm. (**E**) The expression level of MAPKs. 5 mM glutamate increases p-P38, p-ERK, and p-JNK, but co-treatment of EEPG decreases the level of p-P38, p-ERK, and p-JNK. (**F**–**H**) Bar graphs representing the intensity of immunoblot bands, as quantified using Image J software (Version 1.54k). *** *p* < 0.001, ** *p* < 0.002, and * *p* < 0.02 versus 5 mM glu alone group. ^##^ *p* < 0.01 and ^###^ *p* < 0.001 versus untreated control group.

In order to study whether EEPG affects glutamate-induced increases in cellular Ca^2+^ ion concentration in HT22 cells, we used Fluo-3, a fluorescent Ca^2+^ ion-sensitive dye, to quantify intracellular Ca^2+^ levels. Glutamate increased the intracellular Ca^2+^ ion levels. However, the co-treatment of EEPG (100 μg/mL) with glutamate reduced cellular Ca^2+^ ion concentration to levels similar to those observed with DMSO, as indicated by fluorescent intensity (Figure 2A,B).

Glutamate treatment also increases intracellular ROS levels in HT22 cells. To study the intracellular accumulation of ROS levels, we used 2′,7′-dichlorodihydrofluorescein diacetate (DCF-DA), a fluorescent ROS sensor. Glutamate treatment increased ROS fluorescent intensity, while co-treatment with EEPG and glutamate reduced ROS fluorescence in HT22 cells (Figure 2C,D). Both the Ca^2+^ and ROS assays showed that EEPG treatment prevented intracellular ROS production and the increase in cellular Ca^2+^ ion concentration.

Additionally, we assessed the antioxidant effects of EEPG using the DPPH assay. EEPG demonstrated approximately 25% radical scavenging activity at 100 μg/mL (Figure S2). In the DPPH assay, EEPG showed relatively low antioxidant properties. Therefore, the prevention of ROS by EEPG may not be related to the antioxidant property of EEPG.

Numerous studies have demonstrated that the accumulation of ROS triggers the activation of the MAPK signaling pathway. To explore this further, we observed the levels of phosphorylated MAPK proteins that are closely associated with oxidative stress [17,18,19]. In line with previous findings, glutamate treatment was found to activate MAPK proteins (ERK, p38, and JNK), while EEPG treatment effectively inhibited this activation of the MAPK signaling pathway (Figure 2E–H)

### 2.3. EEPG Blocks Caspase Independent Cell Death Pathway by Inhibiting AIF Translocation to Nucleus

**Figure 3:**
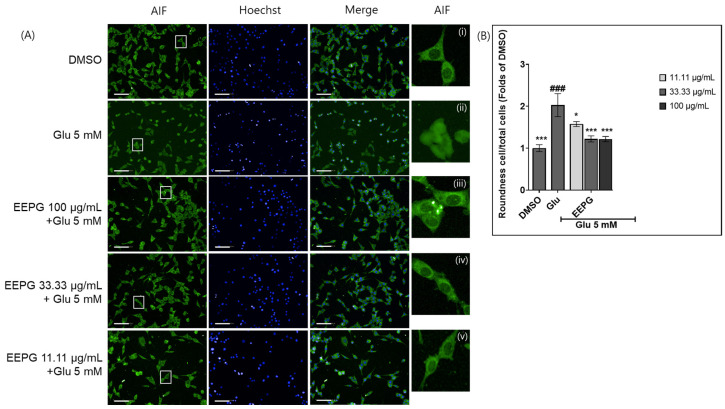

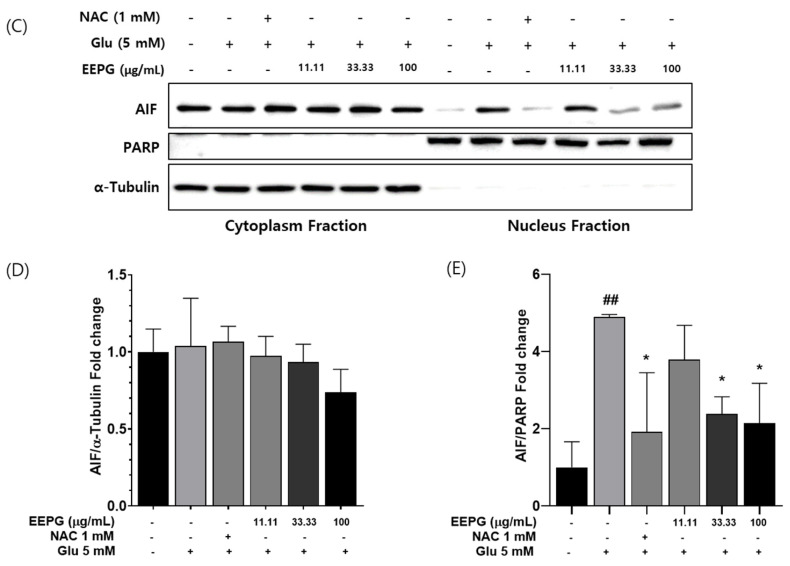
The inhibitory effect of EEPG against AIF translocation to the nucleus in HT22 cells with glutamate-induced cell death. (**A**) Visualization of AIF and nuclei using an AIF antibody and Hoechst dye in HT22 cells. An amount of 5 mM of glutamate induces AIF translocation from mitochondria to the nucleus. Cotreatment of EEPG and 5 mM glutamate inhibits AIF translocation. Scale bars = 100 μm. (i) DMSO, (ii) 5 mM glutamate induced translocation of AIF into the nucleus. (iii) 100 μg/mL (iv) 33.33 μg/mL and (v) 11.11 μg/mL of EEPG decreased translocation of AIF to the nucleus. (**B**) The bar graph indicates the fluorescent intensity of AIF in the nucleus. (**C**) Protein levels of AIF in the cytoplasm and nucleus fraction. (**D**) The bar graphs show AIF levels in the cytoplasm. (**E**) The bar graphs show AIF levels in the nucleus. * *p* < 0.03 and *** *p* < 0.001 versus 5 mM glu alone group. ^##^ *p* < 0.01 and ^###^ *p* < 0.001 versus untreated control group.

Literature reports indicate that glutamate-induced cell death in HT22 cells exhibits characteristics of both apoptosis and necrosis [22,23]. Additionally, studies have shown that glutamate neurotoxicity induces apoptosis via an AIF-dependent pathway [16,24]. We therefore investigated whether EEPG could prevent AIF-mediated apoptosis. Similar to previous reports, glutamate treatment induced the translocation of AIF to the nucleus. Immunohistochemistry with image analysis showed that the accumulation of AIF in the nucleus, as assessed by cell roundness values, decreased with the co-treatment of glutamate and EEPG (Figure 3A,B). Western blot analysis showed an increase in nuclear AIF fraction following glutamate treatment. Conversely, the nuclear fraction of AIF was gradually reduced by co-treatment of EEPG (Figure 3C–E). These data indicate that EEPG treatment inhibits the translocation of AIF to the nucleus from mitochondria in glutamate-induced neuronal cell death.

### 2.4. EEPG Ameliorates Neuroprotection Capability in a Permanent Ischemic Brain Injury Rat Model

**Figure 4:**
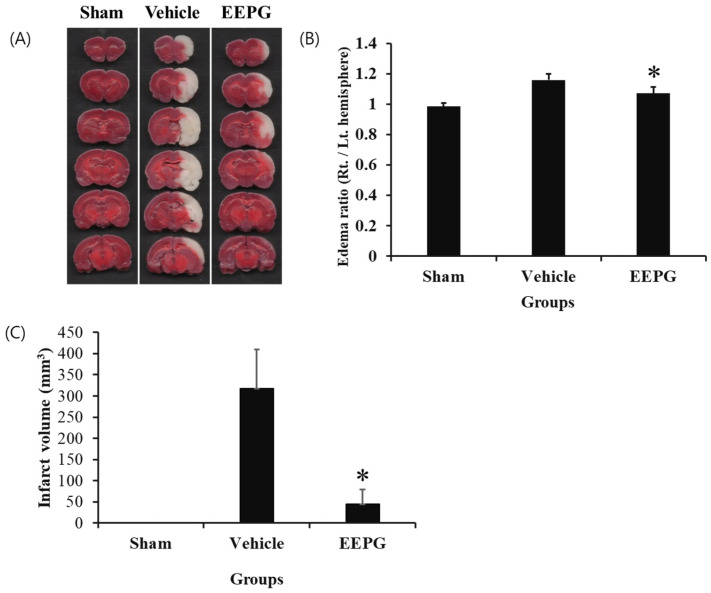
(**A**) The images of 2,3,5-tetraphenyltetrazolium chloride (TTC) staining of the rat brain slices were obtained from the vehicle and EEPG-treated rats. Sham group; no ischemia induction. Vehicle; ischemia induction. EEPG; 30mg/kg of EEPG was administered, followed by the induction of ischemia. (**B**) The comparison of rat brain edea ratio. * *p* < 0.05 (**C**) Quantification of infarct volumes based on TTC staining. Infarct volume of sham, vehicle, and EEPG treated rats. * *p* < 0.005.

The infarct volume resulting from permanent focal ischemia was assessed using TTC staining (Figure 4A). The edema ratio, which is the ratio of the infarcted cerebral hemisphere to the contralateral cerebral hemisphere, was significantly increased by MCAO, but decreased by EEPG administration (Figure 4B). In the sham group, no cerebral infarction was observed in the entire brain. However, in the vehicle group, extensive cerebral infarction areas were identified in the striatum and cerebral cortex. In the EEPG group, the infarct volume was significantly reduced, similar to that observed in the sham group (Figure 4A,C).

Histomorphological and neuronal changes were observed through cresyl violet (CV) staining and Fluoro-Jade C (FJC) in the cerebral cortex and striatum, where cerebral infarctions mainly occurred. In most neurons of the cerebral cortex and striatum of the sham group, Nissl bodies stained with CV and nucleus were clearly observed and appeared to be intact (Figure S3A,B). In the vehicle group, the morphology of neurons in the cerebral cortex and striatum was barely observable, and only small glial cells were seen in CV-stained cells (Figure S3C,D). In the EEPG group, dead and condensed neurons appeared in the striatum and cerebral cortex, but many neurons with Nissl bodies were also observed, showing the neuroprotective effect of EEPG at the histological level (Figure S3E,F).

**Figure 5:**
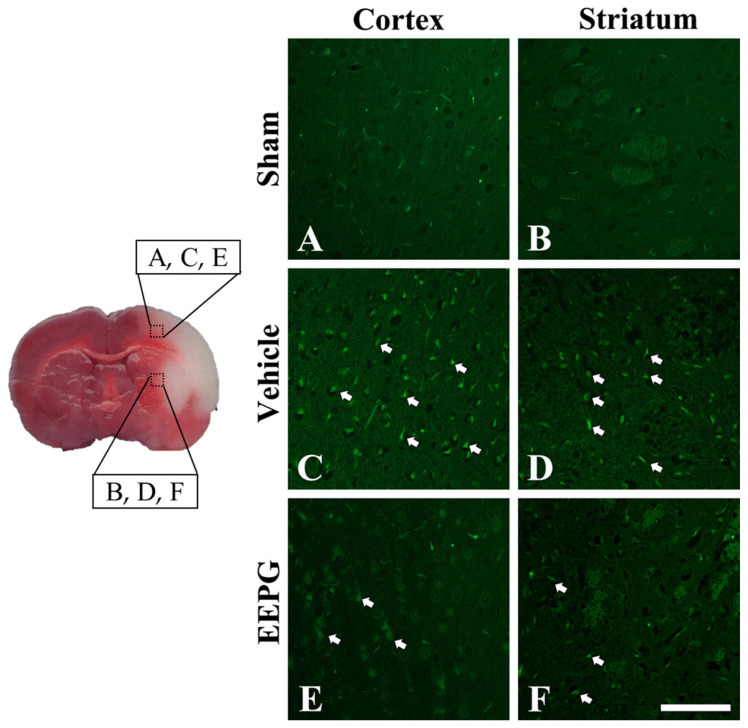
FJC (Fluoro-Jade C) staining in the cortex (**A**,**C**,**E**) and striatum (**B**,**D**,**F**) of the sham (**A**,**B**), vehicle (**C**,**D**) and EEFP (**E**,**F**) at 1 day after MCAO. In the vehicle group, FJC-positive neurons were significantly observed (white arrow). However, the EEPS group exhibited a small number of FJC-positive neurons. Scale bar = 100 μm.

To evaluate the neuroprotective effect against neuronal death due to cerebral infarction, we detected degenerating neurons using FJC staining. The sham group exhibited no fluorescent signal throughout the brain, including the cortex and striatum. In contrast, the Vehicle group displayed a strong fluorescent signal in these regions, indicating significant FJC(+) staining (white arrows in Figure 5), which reflects neuronal cell death following MCAO. Notably, the EEPS group showed reduced FJC(+) staining in the penumbra of the cortex and striatum compared to the Vehicle group, suggesting a neuroprotective effect of EEPS.

### 2.5. HPLC Analysis of EEPG

To identify the constituents of EEPG, we performed rapid isolation of its compounds. A total of 15 compounds were obtained, identified as sterols, flavonoids, etc. Through a comprehensive examination of the physical and spectroscopic data of the purified compounds, the 15 compounds were identified as stigmasterol (1) [21], stigmasterol-3-O-*β*-glucopryanoside (2) [25], adenosine (3) [26], uracil arabinoside (4) [27], afzelin (5) [28], quercetin-3-O-(4″-methoxy)-*α*-L-rhamnopyranoside (6) [29], quercitrin (7) [30], thymidine (8) [31], tamarixetin 3,7-di-O-*α*-L-rhamnopyranoside (9) [21], 3-O-[*β*-D-glucopyranosyl-(1→4)-*β*-glucuronopyranosyl] oleanolic acid 28-O-*β*-D glucopyranosyl ester (10) [31], ladyginoside A (11), polyscioside B (12) [31], quercetin 3,7-dirhamnoside (13) [31], rutin (14) [32], and nicotiflorin (15) [33] (Table S1).

**Figure 6:**
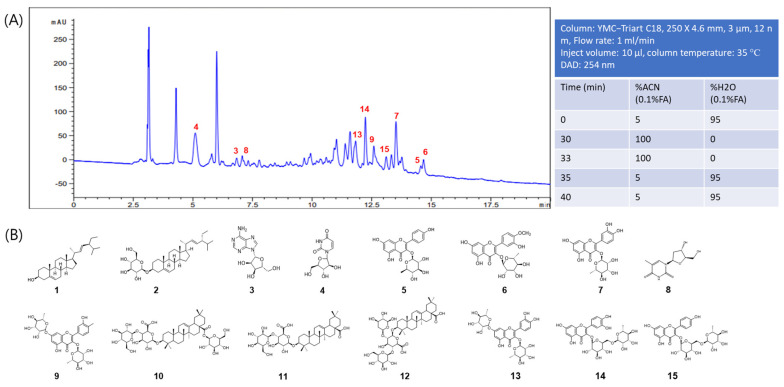
(**A**) HPLC trace of EEPG. HPLC profile was obtained by injection of 10 mg of EEPG. (**B**) The structures of purified compounds in EEPG extract.

HPLC analyses identified 10 compounds with retention times at 254 nm as follows: 5.1 min (compound **4**), 6.84 min (compound **3**), 7.07 min (compound **8**), 11.83 min (compound **13**), 12.24 min (compound **14**), 12.59 min (compound **9**), 13.11 min (compound **15**), 13.52 min (compound **7**), 14.58 min (compound **5**), and 14.67 min (compound **6**) (Figure 6A). The identification was accomplished by analyzing the unknown peak’s absorption spectra and retention data of the extract and comparing them with purified compounds.

### 2.6. Stigmasterol (1) and Stigmasterol-3-O-β-D-glucopyranoside (2) Are the Most Active Neuroprotective Constituents Found in EEPG

**Figure 7:**
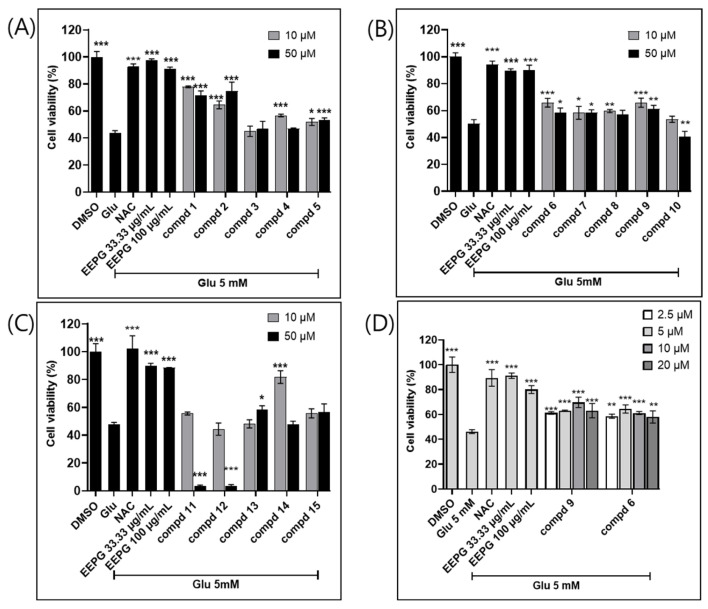
The neuroprotective effects of purified compounds in HT22 cells with glutamate-induced cell death. (**A**–**C**) 15 compounds were evaluated for their neuroprotective effects against glutamate-induced neuronal cell death. Compounds (**1**, **2**, **6**, **9**, and **14**) showed neuroprotective effects, whereas other compounds showed weak or no neuroprotective activity in HT22 cells. (**D**) Among compounds (**1**, **2**, **6**, and **9**), compounds (**6** and **9**) were further evaluated for their neuroprotective effects against glutamate-induced neuronal cell death, and both exhibited moderate neuroprotective effects in HT22 cells. *** *p* < 0.001, ** *p* < 0.01, * *p* < 0.1.

To identify the active compounds in EEPG, we further assessed 15 isolated compounds in HT22 cells for their effects against glutamate neurotoxicity (Figure 7A–C). Among these compounds, stigmasterol (1), stigmasterol-3-O-*β*-D-glucopyranoside (2), rutin (14), Quercetin-3-*O*-(4″-methoxy)-*α*-L-rhamnopyranoside (6), and Tamarixetin 3,7-di-*O*-*α*-L-rhamnopyranoside (9) showed potent neuroprotective effects in glutamate-mediated neurotoxicity in HT22 cells (Figure 7D and Figure S4). Among the active constituents, stigmasterol (1), stigmasterol-3-O-*β*-D-glucopyranoside (2), and rutin (14) have been reported to possess neuroprotective capabilities. Quercetin-3-*O*-(4″-methoxy)-*α*-L-rhamnopyranoside (6), and Tamarixetin 3,7-di-*O*-*α*-L-rhamnopyranoside (9) also showed neuroprotective effect against glutamate neurotoxicity. Cell viability increased by more than 60% with Quercetin-3-O-(4″-methoxy)-*α*-L-rhamnopyranoside (6) and Tamarixetin 3,7-di-O-*α*-L-rhamnopyranoside (9), each at approximately 10 μM concentration. Our focus was on investigating the neuroprotective effects of less commonly recognized compounds, rather than well-reported ones such as stigmasterol (1), stigmasterol-3-O-*β*-D-glucopyranoside (2), and rutin (14).

Therefore, we evaluated the neuroprotective effects of Quercetin-3-O-(4″-methoxy)-*α*-L-rhamnopyranoside (6) and Tamarixetin 3,7-di-O-*α*-L-rhamnopyranoside (9). The cell viability assay demonstrated that treatment with either Quercetin-3-O-(4″-methoxy)-*α*-L-rhamnopyranoside (6) or Tamarixetin 3,7-di-O-*α*-L-rhamnopyranoside (9) increased cell viability by approximately 60% to 70% in glutamate-induced HT22 cell death (Figure 7D). We further demonstrated that AIF translocation to the nucleus is blocked by the treatment of these two compounds. Therefore, we identified that both Quercetin-3-*O*-(4″-methoxy)-*α*-L-rhamnopyranoside (6) and Tamarixetin 3,7-di-*O*-*α*-L-rhamnopyranoside (9) showed neuroprotective effects in HT22 cells (Figure S5A,B).

## 3. Discussion

*P. guilfoylei* is distributed in Eastern Asia and the Pacific region. Unlike *Polyscias fruticosa*, the medicinal properties of *Polyscias guilfoylei* have not been extensively reported. Although there are a few reports indicating that components of *Polyscias guilfoylei* possess anti-inflammatory and cytotoxic effects, research has yet to explore the neuroprotective potential of *Polyscias guilfoylei* extract in relation to glutamate-induced cell death in HT22 cells or in permanent brain ischemia models in rats. HT22 cells are used to observe oxidative stress effects induced by glutamate treatment. Glutamate treatment causes oxidative stress, leading to neuronal cell death by increasing cellular ROS and Ca^2+^ ion concentrations. Previous reports indicate that oxidative stress induces mitochondrial dysfunction, which can lead to AIF-mediated apoptotic cell death, a caspase-independent apoptotic pathway. Therefore, HT22 cell death induced by glutamate serves as a viable cell-based assay model for identifying extracts and compounds with neuroprotective potential.

Here, we have identified the neuroprotective effects of the ethanol extract of *Polyscias guilfoylei* (EEPG) in HT22 cells. EEPG was shown to protect against glutamate neurotoxicity in HT22 cells. Specifically, EEPG attenuated the increase in intracellular Ca^2+^ ion levels and ROS generation caused by glutamate treatment. It also inhibited the translocation of AIF from mitochondria to the nucleus and blocked MAPK activation. Furthermore, we demonstrated the protective potential of EEPG in permanent brain ischemic models. We isolated individual constituents from EEPG and evaluated their neuroprotective effects against glutamate. Among these constituents, stigmasterol (1), daucosterol (2), and rutin (14) exhibited neuroprotective effects consistent with previous reports. In contrast, quercetin-3-O-(4″-methoxy)-*α*-L-rhamnopyranoside (6) and tamarixetin 3,7-di-O-*α*-L-rhamnopyranoside (9) showed relatively lower neuroprotective effects and their neuroprotective properties have not been previously reported.

## 4. Material and Methods

### 4.1. Plant Materials and Extraction

Dried leaves of *Polyscias guilfoylei* (4 kg) were extracted using 70% ethanol (24 L) over 3 days at room temperature. The obtained supernatant was then filtered using filter paper (Whatman, Buckinghamshire, UK). This extraction process was repeated three more times with the remaining powders. The combined liquids from these extractions were evaporated at 45 °C using a Rotavapor R-100 (Büchi, Flawil, Switzerland), yielding a 350 g residue of extract. The ethanol extract of *Polyscias guilfoylei* (EEPG) was stored at −20 °C.

### 4.2. Isolation of Single Compounds and Structure Identification

The EEPG was first dissolved in water and subsequently fractionated using hexane (Hx), ethyl acetate (EA), and butanol (Bu) to obtain the Hx fraction (16 g), EA fraction (30 g), and Bu fraction (25 g). The EA fraction was added into a silica gel column and eluted with a stepwise gradient of Hx-EA (100:1 to 0:1) and DCM (dichloromethane)-MeOH (20:1 to 1:1) to obtain 20 subfractions (PGE 1.1–PGE 1.20). Compound **1** (33.2 mg) and compound **2** (80.3 mg) were recrystallized from PGE 1.3 and PGE 1.13, respectively. Subfractions PGE 1.13 and PGE 1.15 were selected for further isolation using silica gel RP-C18 and purified by preparative MPLC, yielding compound **5** (3.6 mg) and compound **6** (1.2 mg) from PGE 1.13, and compound **3** (1.5 mg), compound **4** (1.5 mg), compound **7** (1.5 mg), and compound **8** (1.5 mg) from PGE 1.15 (Figure 6).

The Bu fraction was also applied to a column made of silica gel and purified with a stepwise gradient of DCM-MeOH (100:1 to 0:1), generating 13 subfractions (PGB 1.1–PGB 1.13) in which, subfraction PGB 1.10 and PGB 1.13 were chosen for further isolation by using Diaion resin HP-20, Silica gel RP-C18, Sephadex LH-20 column and purified by preparative MPLC to yield compound **9** (3.0 mg) from PGB 1.10; compound **10** (20.1 mg), compound **11** (10.6 mg), compound **12** (10.3 mg), compound **13** (1.5 mg), compound **14** (3.3 mg), and compound **15** (3.4 mg) from PGB 1.13 (Figure 6).

### 4.3. HPLC Profiling of EEPG

The experiments utilized a liquid chromatography system Agilent Series 1200 and a YMC Triart C18 column and it was maintained at 35 °C. Figure 6A shows further details of the HPLC procedure. The peak shapes were refined with 0.1% formic acid in acetonitrile and water. The injection volume was set to 10 µL at a speed of 1 mL/min, and a 254 nm UV sensor was used for detection. For HPLC analysis, EEPC 10 mg was suspended in methanol, and an aliquot was used.

### 4.4. HT22 Cell Culture

The HT22 cells were purchased from the Korea cell line bank (KCLB) and it was grown using DMEM high glucose media with 10% heat inactive FBS and 1% penicillin/streptomycin under 5% CO_2_ at 37 °C [34].

### 4.5. Cell Viability Assay

HTT22 cells (3000 per well) were added to 96-well plates and incubated at 37 °C with 5% CO_2_ for 12 h. The control (0.5% DMSO) and extracts at indicated concentrations were treated and incubated for 2 h. Then, glutamate (5 mM) was treated in each well. The cells were incubated for an additional 12 h before being stained with 1 µM Calcein AM-containing media. Cell viability was assessed using the Operetta image analysis system (PerkinElmer, Waltham, MA, USA). Alternatively, after the incubation of 12 h, cell viability was measured with the addition of EZ-Cytox (DoGenBio, Seoul, Republic of Korea) reagent. Then, absorbance was measured at a wavelength of 450 nm.

### 4.6. Intracellular ROS and Ca^2+^ Influx Assay

HTT22 cells (3000 per well) were seeded in 96-well plates and incubated at 37 °C with 5% CO_2_ for 12 h. EEPG at concentrations of 100, 33.33, and 11.11 µg/mL was treated in each well as pretreatment, and after 2 h glutamate 5 mM was treated further. Then, cells were incubated for 8 h for ROS generation. The media were removed and 10 µM DCFDA (2′,7′-dichlorodihydrofluorescein diacetate) was treated and incubated for 30 min. Then, wells were washed three times with DPBS, and images were captured and analyzed using the operetta system.

For the measurement of intracellular Ca^2+^ ion levels, we used the Fluo-3 fluorescent probe, following the same procedure as for cellular ROS measurement. To measure intracellular Ca^2+^ ion levels, 2 μM of Fluo-3 AM fluorescent probe was added to each well for 15 min, followed by washing with DPBS and analysis using the Operetta system.

### 4.7. Immunoblot Analysis

HT22 cells were treated with the extract and glutamate for 12 h. After treatment, the cells were harvested and lysed in CEB buffer (Invitrogen, Waltham, MA, USA), prepared with the addition of a protease inhibitor cocktail, for 30 min. The lysates were centrifuged at 14,000 rpm for 20 min at 4 °C. The protein supernatant was collected and quantified using the BCA assay. Proteins were then separated by SDS-PAGE at 100 V and transferred onto PVDF membranes at 100 V for 90 min. The membranes were incubated with primary and secondary antibodies, and immunoblot signals were detected using chemiluminescence and visualized with the LAS4000 gel documentation system (FujiFilm, Tokyo, Japan)

### 4.8. In Vivo Efficacy Test of Extracts

#### 4.8.1. Experimental Animals

For the experiment, 7-week-old male Sprague Dawley rats (Orient Bio, Republic of Korea) weighing between 260 g and 280 g were used. They were maintained at 12 h light and dark cycle at 23 °C and 60% humidity. The rats had unrestricted access to water and food. Animal experiments were performed by following the animal ethics guidelines of ARRIVE and national institutes of health. All the experiment procedures were reviewed by the IACUC (Institutional Animal Care and Use Committee) of Gangneung-Wonju National University (Approval No. GWNU2023-15).

#### 4.8.2. Pre-Treatment with EEPG

To investigate the neuroprotective effects of EEPG in an in vivo animal model, Sprague Dawley (SD) rats were divided into three groups, with seven rats per group: the sham group, the vehicle group, and the EEPG-treated group. All groups underwent ischemic surgery, except for the sham group. Rats in the EEPG-treated group received 30 mg/kg of EEPG, which was dissolved in cell culture-grade water, serving as the vehicle. EEPG and the vehicle were administered intragastrically using a feeding needle once daily for 7 days prior to the ischemic surgery.

#### 4.8.3. Permanent Focal Cerebral Ischemia

The 3% isoflurane was used to sedate rats and 2% isoflurane was maintained throughout the surgery. A rectal thermometer was used to monitor body temperature, and temperature was maintained at 37 °C. Focal cerebral ischemia was induced using an adapted version of the occlusion model developed by Kao et al. [35]. A midline cervical incision was performed, followed by careful dissection of the nerves and fascia to expose the right common carotid artery. The peripheral carotid artery and common carotid artery were completely ligated with a 5-0 silk stitch. The internal artery was loosely occluded with a 5-0 silk stitch. A small incision was performed at the bifurcation of the carotid artery, and a monofilament 4-0 nylon suture coated with dental silicone was inserted 17 mm into the internal carotid artery and secured with a 5-0 silk suture. The neck incision was then closed with a 4-0 silk suture. After recovering from anesthesia, the rat was returned to cages.

#### 4.8.4. TTC Staining and Quantification of Ischemic Infarction

Twenty-four hours after the middle cerebral artery occlusion, chloral hydrate (400 mg/kg, i.p.) was used to sedate the rats. The head was decapitated using a guillotine, the skull was carefully removed, and quickly brain was extracted. The brain was positioned on a slicing frame (rat brain matrix, ASI Instruments, Warren, MI, USA), sliced into 2 mm thick sections using a razor, and stained with 2% 2,3,5-triphenyltetrazolium chloride (TTC, Sigma, Inc., Burlington, MI, USA) for 15 min. After staining, a 4% PFA (paraformaldehyde) solution was used to fix the tissue sections.

Brain tissue stained with TTC was imaged with a scale bar using a scanner (Epson, Suwa, Nagono, Japan), and the infarct volume and total brain volume in each section were corrected with the scale bar using Image J (NIH, Bethesda, MD, USA) to obtain the area. The brain edema index was calculated using the volume of both cerebral hemispheres, and the corrected cerebral infarction area was calculated using this value. The cerebral infarction volume was expressed numerically as the sum of the volumes measured in six 2 mm thick brain slices.

The data shown here represent the means ± SD. The difference between the means of the Vehicle group and the EEPG group was statistically analyzed using the nonpaired Student’s *t* test in order to compare the effectiveness of EEPG against ischemic damage. Statistical significance was considered at *p* < 0.05.

#### 4.8.5. Tissue Processing and Staining

The fixed brain sections were embedded in paraffin blocks following the standard dehydration and paraffinization process. Then, using a microtome (Leica, Wetzlar, Hesse, Germany), the paraffin blocks were sliced into 5 μm sections and placed on saline-coated slide glasses for tissue staining.

Cresyl violet staining: Tissue sections that had been deparaffinized and hydrated were stained with a 0.1% cresyl violet acetate (Sigma, Burlington, MI, USA) solution. The stained tissues were then quickly rinsed with distilled water, differentiated in 95% ethanol for 10 min, dehydrated twice in ethanol (100%) for 5 min each, cleared twice in xylene for 5 min each, and finally mounted with Canada balsam (Kanto, Tokyo, Japan).

FJC staining: We followed a previously published procedure using 0.0001% Fluoro-Jade C (Millipore, Burlington, MA, USA) in a 0.1% acetic acid solution. All reactions were conducted at room temperature.

## 5. Conclusions

In our study, we demonstrated the neuroprotective effects and underlying mechanisms of *Polyscias guilfoylei* in a glutamate-induced neuronal cell death model using HT22 cells. Additionally, we showed that EEPG exhibited therapeutic potential in rat models of permanent brain ischemia. We believe that this study highlights the potential of herbal medicine as a treatment for brain ischemia.

## Data Availability

All the data are provided within the article and Supporting Information Data will be shared upon request (Jae Wook Lee, Institute of Natural Products, Korea Institute of Science and Technology, jwlee5@kist.re.kr).

## Supplementary Materials

The following supporting information can be downloaded at https://www.mdpi.com/article/10.3390/ijms252212153/s1.
