# Supplementary material for: Neuroprotective Effects of Ethanol Extract Polyscias guilfoylei (EEPG) Against Glutamate Induced Neurotoxicity in HT22 Cells
Source: Int J Mol Sci. 2024 Nov 12;25(22):12153. doi: 10.3390/ijms252212153 (PMC11595212; doi:10.3390/ijms252212153)
Supplement: Supplementary file 1 [file ijms-25-12153-s001.zip › ijms-3261645-supplementary.pdf]

Supporting Information

Neuroprotective Effects of Ethanol Extract *Polyscias guilfoylei* (EEPG) Against Glutamate Induced Neurotoxicity in HT22 Cells

Qui Ngoc Sang Nguyen [1,2,3,†], Ki-Yeon Yoo [2,†], Thi Thu Trang Pham [1,4], Baskar Selvaraj [1], Huong Thuy Vu [5,6], Tam Thi Le [1], Heesu Lee [2], Quang Luc Tran [5], Phuong Thien Thuong [7], Ae Nim Pae [8,9], Sang Hoon Jung [1,4,*] and Jae Wook Lee [1,4,*]

[1] Natural Product Research Center, Institute of Natural Products, Korea Institute of Science and Technology, Gangneung 25451, Republic of Korea; kevindh2515@kist.re.kr (Q.N.S.N.); 523502@kist.re.kr (T.T.T.P.); sbaskar@kist.re.kr (B.S.); ttle@kist.re.kr (T.T.L.)
[2] Department of Anatomy, College of Dentistry and Research Institute for Dental Engineering, Gangneung Wonju National University, 7 Jukheon-gil, Gangneung 25457, Republic of Korea; kyyoo@gwnu.ac.kr (K.-Y.Y.); nightsu@gwnu.ac.kr (H.L.)
[3] Institute of Natural Product Chemistry, Vietnamese Academy Science and Technology, 1H Building, 18 Hoang Quoc Viet Street, Cau Giay, Hanoi 100000, Vietnam
[4] Natural Product Applied Science, KIST School, University of Science and Technology (UST), Gangneung 25451, Republic of Korea
[5] Traphaco Join-Stock Company, 75 P. Yên Ninh, Quán Thánh, Ba Đình, Hanoi 1000000, Vietnam; vhthuy111@gmail.com (H.T.V.); luctq@traphaco.com.vn (Q.L.T.)
[6] Faculty of Herbal Medicine, Traditional Pharmacy, Hanoi University of Pharmacy, 13-15 Le Thanh Tong, Ba Dinh, Hanoi 100000, Vietnam
[7] Division of Biotechnology, Vietnam Korea Institute of Science and Technology, Hoa lac High-tech Park, km29 Thang Long Boulevard, Hanoi 100000, Vietnam; ptthuong.vkist@gmail.com
[8] Division of Bio-Medical Science & Technology, KIST School, Korea University of Science and Technology (UST), Seoul 02792, Republic of Korea; anpae@kist.re.kr
[9] Center for Brain Disorders, Brain Research Institute, Korea Institute of Science and Technology, Seoul 02792, Republic of Korea
* Correspondence: shjung@kist.re.kr (S.H.J.); jwlee5@kist.re.kr (J.W.L.)
† These authors contributed equally to this work.

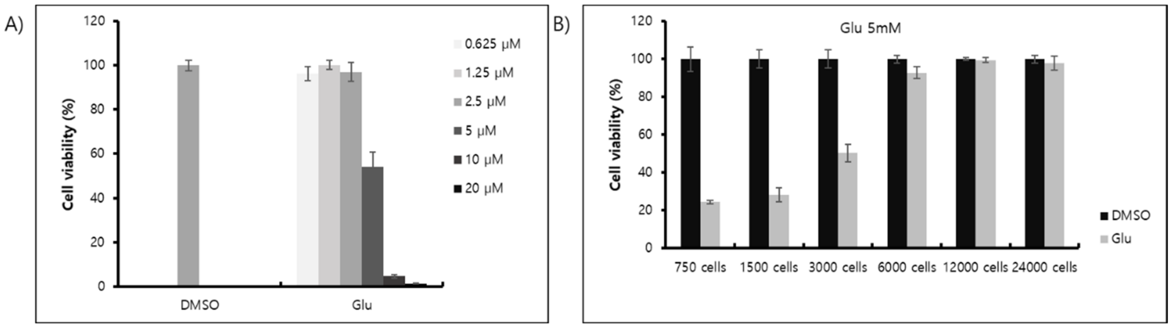

Figure S1. Cell viability assay of glutamate induced cell death in HT22 cells. A) indicated concentration of glutamate was treated in HT22 cells. B) cell survival of HT22 after treatment of 5 mM glutamate in the various cell number of 96 wells.

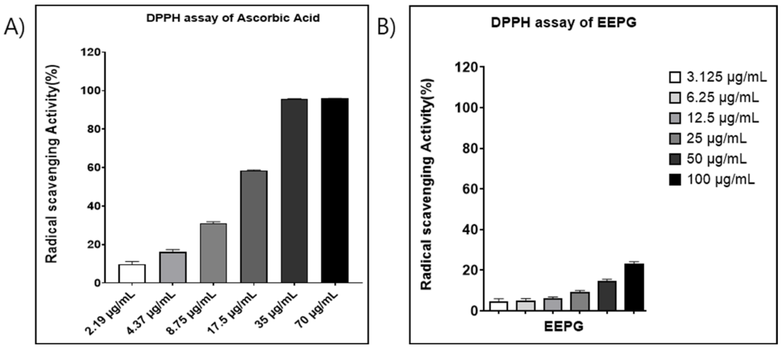

Figure S2. The comparison of antioxidant effect of EEPG using DPPH assay. A) ascorbic acid was used positive control. B) antioxidant effect of the indicated concentration of EEPG.

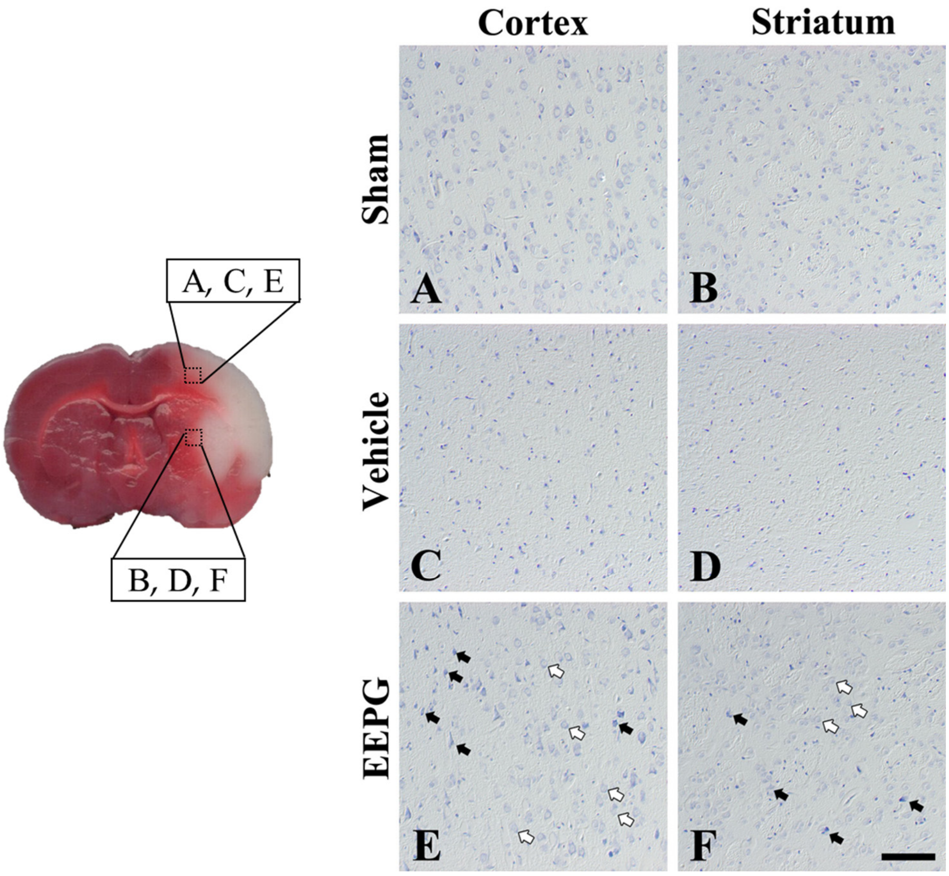

Figure S3. CV (Cresyl Violet) staining in the cortex (A, C and E) and striatum (B, D, and F) of the sham (A and B), vehicle (C and D) and EEFP (E and F) at 1 day after MCAO. In the vehicle group, CV-positive neurons were rarely observed. However, in the EEPG group, a significant number of CV-positive neurons were seen. Among these, a mixture of condensed neurons undergoing apoptosis (black arrows) and normal neurons (white arrows) was observed. Scale bar = 50 μm.

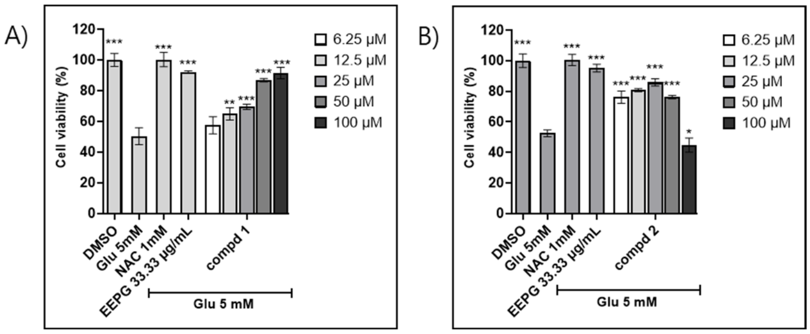

Figure S4. A) The neuroprotective effects of compd 1 (stigmasterol) and B) compd 2 (stigmasterol-3-O-*β*-D-glucopyranoside). ***P<0.001, **P< 0.003, *P<0.06 compared with 5 mM glutamate.

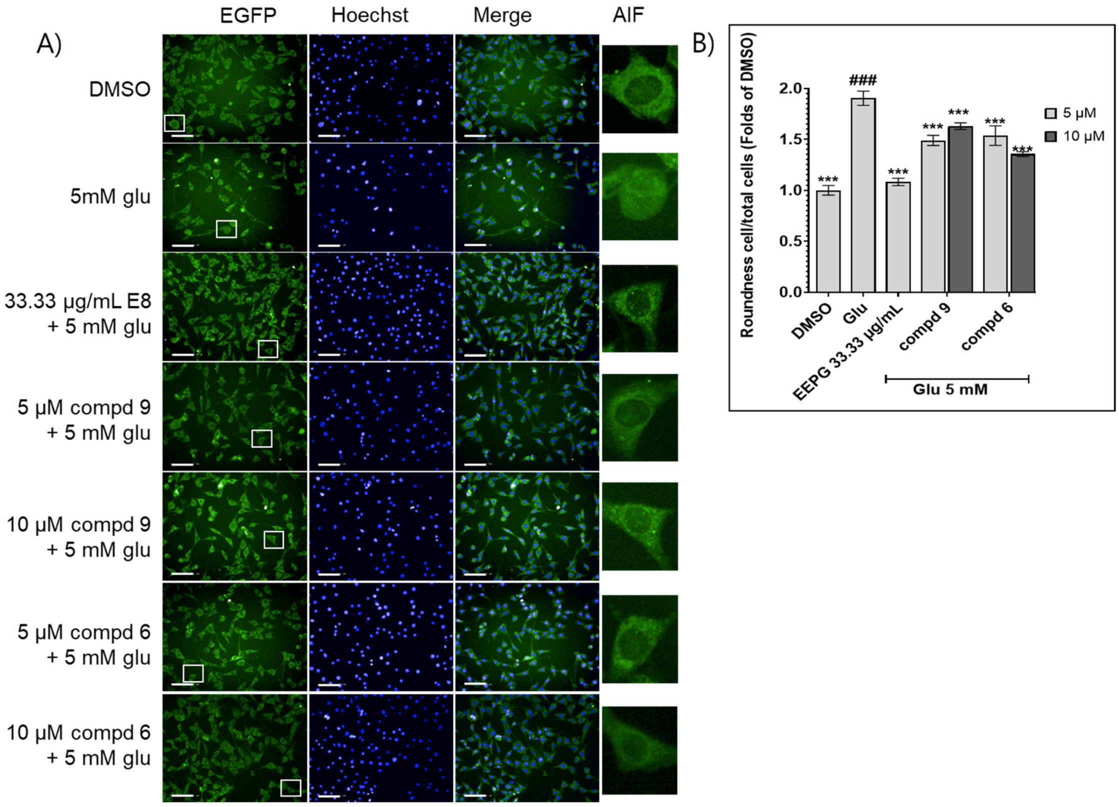

Figure S5. Images of AIF and nuclei using anti-AIF antibody and Hoechst dye in HT22 cells. A) 5 mM of glutamate induces AIF translocation from mitochondria to nucleus. Treatment of compounds 6 or compound 9 inhibit AIF translocation to nucleus. Scale bars = 100 μm B) The bar graph indicates the fluorescent intensity of AIF in nucleus. ***P<0.001 versus 5 mM glu alone group, ###P<0.001 versus untreated control group.

Table S1. The list of isolated compounds from extract of EEPG. Individual compounds were obtained by purification of 350 g of EEPG.

No	Name		Formula	MW (g/mol)	Weight (mg)
1	compound 1	stigmasterol	$C_{29}H_{48}O$	412.70	33.2
2	compound 2	stigmasterol-3-O-*β*-D-glucopyranoside	$C_{35}H_{58}O_6$	574.84	80.0
3	compound 3	adenosine	$C_{10}H_{13}N_5O_4$	267.25	1.5
4	compound 4	uracil arabinoside	$C_9H_{12}N_2O_6$	244.20	1.5
5	compound 5	afzelin	$C_{21}H_{20}O_{10}$	432.38	3.6
6	compound 6	quercetin-3-*O*-(4″-methoxy)-*α*-L-rhamnopyranoside	$C_{22}H_{22}O1_1$	462.41	1.2
7	compound 7	quercitrin	$C_{21}H_{20}O_{11}$	448.38	2.0
8	compound 8	thymidine	$C_{10}H_{14}N_2O_5$	242.23	3.5
9	compound 9	tamarixetin 3,7-di-*O*-*α*-L-rhamnopyranoside	$C_{28}H_{32}O_{15}$	608.55	3.0
10	compound 10	3-O-[*β*-D-glucopyranosyl-(1→4)-*β*-glucuronopyranosyl] oleanolic acid 28-O-*β*-*D* glucopyranosyl ester	$C_{48}H_{76}O_{19}$	957.12	20.2
11	compound 11	ladyginoside A	$C_{42}H_{66}O_{14}$	794.98	10.3
12	compound 12	polyscioside B	$C_{47}H_{74}O_{18}$	927.09	10.1
13	compound 13	quercetin 3,7-dirhamnoside	$C_{27}H_{30}O_{15}$	594.52	1.2
14	compound 14	rutin	$C_{27}H_{30}O_{16}$	610.52	3.4
15	compound 15	nicotiflorin	$C_{27}H_{30}O_{15}$	594.52	3.3
